# Effects of intraguild interactions on *Anticarsia gemmatalis* and *Chrysodeixis includens* larval fitness and behavior in soybean

**DOI:** 10.1002/ps.6330

**Published:** 2021-03-10

**Authors:** Sabrina Ongaratto, Edson LL Baldin, Thomas E Hunt, Débora G Montezano, Emily A Robinson, Maria C dos Santos

**Affiliations:** ^1^ Department of Crop Protection, School of Agriculture São Paulo State University Botucatu São Paulo Brazil; ^2^ Haskell Agricultural Laboratory University of Nebraska‐Lincoln Concord NE USA; ^3^ Department of Entomology University of Nebraska‐Lincoln Lincoln NE USA; ^4^ Department of Statistics University of Nebraska‐Lincoln Lincoln NE USA

**Keywords:** soybean pest, interspecific competition, intraspecific competition, integrated pest management

## Abstract

**BACKGROUND:**

Crop pest management requires an understanding of the complex interactions among species that potentially impact crop yield. In soybean, the velvetbean caterpillar, *Anticarsia gemmatalis* (Hübner), and the soybean looper, *Chrysodeixis includens* (Walker), are described as key pests, sharing the same feeding guild. We assessed the intraguild interactions of these species under laboratory conditions. Fitness cost study was conducted to examine the influence of competition on insect development. A video tracking system was used to evaluate behavioral parameters during larval interactions in scenarios with and without food availability.

**RESULTS:**

In the fitness cost assay, pupal weight was not significantly affected, regardless of sex. However, larval and pupal survival were influenced by the competition, especially in third *versus* fifth instar scenarios. We detected 40.00% cannibalism and 46.67% predation when *A. gemmatalis* and *C. includens* third instars competed with *A. gemmatalis* fifth instar, respectively. Distance moved, distance between larvae, body contact (food available) and frequency in food of *C. includens* larvae were negatively affected by interactions. *Anticarsia gemmatalis* larvae showed highly active behavior, moving twice or more the distance compared to *C. includens* larvae, and *A. gemmatalis* spent more time in body contact with food.

**CONCLUSION:**

Our results suggest that *A. gemmatalis* has a competitive advantage over *C. includens*. This study provides important information regarding lepidopteran behavior in soybean. We recommended that additional studies are necessary to understand the effects of interactions, especially in field conditions.

© 2021 The Authors. *Pest Management Science* published by John Wiley & Sons Ltd on behalf of Society of Chemical Industry.

## Graphical abstract

*Anticarsia gemmatalis* has a competitive advantage over *Chrysodeixis includens*. Our findings provide significant information regarding lepidopteran development and behavior, contributing to the development of integrated pest management and resistance management strategies

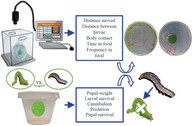

## 1 INTRODUCTION

Soybean [(*Glycine max* (L.) Merrill)] is one of the most important agricultural products and has been increasingly planted worldwide.^1^, ^2^ Despite the diverse climate conditions, important soybean producing areas in USA and South American countries are threatened by lepidopteran caterpillars.^3^, ^4^, ^5^ The velvetbean caterpillar, *Anticarsia gemmatalis* (Lepidoptera: Erebidae), and the soybean looper, *Chrysodeixis includens* (Lepidoptera: Noctuidae), are among the major soybean pests, which have been causing economic damage from South to North America.^6^, ^7^, ^8^

The management of both lepidopteran species can be achieved using different control strategies as part of integrated pest management (IPM) programs, but recently in Brazil, genetically modified soybean expressing a *Bacillus thuringiensis* (*Bt*) insecticidal protein has assumed a prominent position for large scale management of lepidopteran insect pests.^9^, ^10^ Although this biotechnology is an important tool for the integrated management of *A. gemmatalis* and *C. includens*, the use of *Bt* transgenic crops has been modifying Lepidoptera entomofauna in soybean.^9^, ^11^, ^12^ In the US there is no *Bt* transgenic soybean; however, taking it as an example, the large scale adoption of *Bt* corn and cotton in the US has resulted in an intense selection pressure for the development of resistance and challenges the long‐term sustainability of the technology.^13^ Thus, how can studying interactions between insects help manage the soybean production system? In general, intra and interspecific competition dynamics among phytophagous species that are closely related are major ecological processes which shape the patterns of these species abundance, distribution, and diversity.^14^, ^15^ However, despite the strong evidence that herbivore competition is ubiquitous, the vast majority of investigations focus on single leaf‐chewing or grain‐feeding species,^16^, ^17^, ^18^ leaving the competitive interactions between leaf‐eaters as a largely unexplored subject.

Several aspects should be considered when examining interaction of different caterpillar species. Intraguild competition has complex functions that work at any level from individual fitness to community structure pattern, being affected for many reasons.^19^ Among the consumptive effects, cannibalism and predation may intensify intraguild competition. Cannibalism may result in population self‐regulation, while predation among individuals of competing species tends to ‘regulate’ the total biomass of individuals.^20^, ^21^ In this sense, competitive movements are particularly important for the acquisition of and/or defense of limited resources, allowing insects to survive and continue development and pass their genes to the next generations. Studies indicate that for many species the genetic background of the insects is involved with competitiveness among species, including in relation to competitive behavior. Finally, the widespread occurrence of various adaptations that either inhibit or promote aggressive behavior among relatives implies the presence of selection on genes that regulate its expression.^22^

Mutually or alternatively, the direction of competitive interactions may not have simply been due to direct effects, but also/instead to indirect competitive effects, such as resource competition/depletion, avoidance, exclusion, and can be a response to many environmental factors. Additional factors can also increase activity and exposure to non‐conspecific predation (e.g., biological control), tradeoffs of activity on non‐mass/survival elements of fitness (e.g., reproduction), especially under field conditions. Life history and physiological condition of an individual and the ecological and social conditions could also modify the likelihood of these kinds of behavior.^20^, ^21^ All of these factors, combined or not, would lead to the consequence of larval intraguild interaction impacting aggressive behavior and fitness.

In this context, the behavior of lepidopterans exposed to insecticides has been explored in order to understand the influence on fitness^23^ and on the efficacy of pest management.^24^ In addition, some studies have investigated how pest behavioral responses to insect‐resistant transgenic crops are influenced by alleles that confer resistance to plant‐incorporated toxins,^25^, ^26^, ^27^, ^28^, ^29^, ^30^ and just a few studies have been designed to understand the feeding behavior^31^ and the intraguild interactions of different lepidopteran species in corn^32^, ^33^ and cotton.^34^ In soybean, there are only competition studies involving stinkbugs.^35^ Thus, further research involving other species, such as *A. gemmatalis* and *C. includens*, is highly desirable.

Considering the importance of soybean and the expansion of *Bt* crop cultivation, a better understanding of the behavior and interaction between insect species has become increasingly necessary and can help in the management of *A. gemmatalis* and *C. includens*. Thus, the objective of this study was to evaluate the intraspecific and interspecific interactions of *A. gemmatalis* and *C. includens* larvae on soybean in different competition scenarios under laboratory conditions, and its effects on fitness. We also assessed the larval interactions using an automated video tracking system to describe details of larval behavior in scenarios in the presence and absence of food, under controlled conditions.

## 2 MATERIALS AND METHODS

### 2.1 Insects

For the fitness cost assay, colonies of *A. gemmatalis* and *C. includens* were kept under laboratory conditions [(25 ± 2°C, RH: 60 ± 10%; 14:10 (L:D)] in the Department of Crop Protection, São Paulo State University, Botucatu, São Paulo, Brazil. The larvae were reared on artificial diet proposed by Greene *et al*,^36^ and additional details on rearing methodology per Parra.^37^ To maintain colony vigor, insects were frequently collected from the field and from the other colonies, identified, and transferred to the specific colonies used in this study.

To provide insects for the video tracking assay, larvae were commercially acquired (Benzon Research Inc., Carlisle, PA) and reared in plastic cups containing 15 mL of artificial diet (based on diet developed by USDA, Stoneville, MS and University of Georgia, Athens, GA). The insects were kept in a rearing chamber [25 ± 2°C, RH 60 ± 10%, 14:10 (L:D)] until the fourth instar.

### 2.2 Fitness cost evaluation

The intraguild interaction assay was performed in competition scenarios involving the two species under laboratory conditions at São Paulo State University, Botucatu, São Paulo, Brazil. Before testing, two larvae from each combination, based on scenarios in Table 1, were taken separately from the colony and starved for 2 h. After that, larvae were placed into transparent plastic cups (100 mL) containing soybean leaves collected from plants at phenological stage V4/V5,^38^ which were maintained in a greenhouse and free from insect infestation. Non‐*Bt* soybean seeds (TMG 7262 RR) were sown in 5 L pots with sterilized soil to provide vegetative tissue for the scenarios.

**Table 1:** Scenarios of intraguild competition involving *A. gemmatalis* and *C. includens* larvae of different sizes for fitness assay

Intraguild competition	
Treatments*	*A. gemmatalis* (third) *versus C. includens* (third)
	*A. gemmatalis* (third) *versus C. includens* (fifth)
	A. gemmatalis (fifth) *versus C. includens* (third)
	*A. gemmatalis* (fifth) *versus C. includens* (fifth)
	*A. gemmatalis* (third) *versus A. gemmatalis* (third)
	*A. gemmatalis* (third) *versus A. gemmatalis* (fifth)
	*A. gemmatalis* (fifth) *versus A. gemmatalis* (fifth)
	*C. includens* (third) *versus C. includens* (third)
	*C. includens* (third) *versus C. includens* (fifth)
	*C. includens* (fifth) *versus C. includens* (fifth)

* Adapted from Dorhout and Rice and Bentivenha *et al* ^29^, ^32^ .

Each plastic cup was considered one replicate, with 15 replicates per scenario. Larvae of the same age were held singly in plastic containers and fed with the same food source. Surviving larvae from the different competition scenarios were kept isolated in the same plastic containers and with the same food source until pupation. When the pupae were 2 days old, individuals were sexed and weighed on an analytical scale (model AY 220 0.0001G, Shimadzu Corporation, Kyoto, Japan) and maintained until adult emergence to verify pupal survival.

### 2.3 Video tracking trials

Automated video tracking software (Ethovision XT 14; Noldus Information Technology, Wageningen, The Netherlands) was used to examine potential differences in behavior between *A. gemmatalis* and *C. includens* when in competition scenarios. The experiment was conducted at the University of Nebraska, Entomology Department, Lincoln, NE, USA. Non‐*Bt* soybean seeds (Pioneer® P25A27X) were sown in 5 L pots with sterilized soil and fertilizer (Peter's 20‐10‐20 general purpose fertilizer/200 ppm Nitrogen) to provide vegetative tissue for the scenarios that offered food. The soybean leaves were collected from plants at phenological stage V4/V5,^38^ which were maintained in a greenhouse free from insect infestation. Larvae were taken separately from plastic cups with artificial diet and starved for 2 h. For each bioassay replication, a pair of larvae were confined together on opposite sides of a petri dish (8 cm diameter × 1.5 cm height; Fisher Scientific, Pittsburgh, PA) with or without a soybean leaf disk (2.5 cm diameter) as a food source, which was classified as food available or food not available. To keep the soybean tissue moist, one layer of solidified agar (2.5% wt:vol, 1.5 mm thickness) was prepared as previously described,^39^ and the leaf tissue was deposited above the layer.

Larvae interactions were assessed in five scenarios (Table 2) with and without food (10 scenarios in total). Each scenario had 15 replications, totaling 150 Petri dishes, and was recorded with a Dino‐Lite AD413T‐12 V camera (Big C, Torrance, CA). Ethovision software was used to collect information on the interactions of the larvae over a 20‐min period. Measurements on each individual larva were taken for the distance moved (cm). When food was present, measurements on each individual larva were taken for frequency in food (n) and time in food (s). For scenarios where two larvae were present, measurements for the distance between larvae (cm) and body contact (s) was also recorded and analyzed.

**Table 2:** Scenarios of intraguild interaction involving *A. gemmatalis* and *C. includens* in the presence or absence of food for the video tracking assay

Intraguild interactions	
Food	*A. gemmatalis* (fourth) *versus A. gemmatalis* (fourth)^a^
	*C. includens* (fourth) *versus C. includens* (fourth)
	*A. gemmatalis* (fourth) *versus C. includens* (fourth)
	*A. gemmatalis* (isolated)
	*C. includens* (isolated)
No food	*A. gemmatalis* (fourth) *versus A. gemmatalis* (fourth)
	*C. includens* (fourth) *versus C. includens* (fourth)
	*A. gemmatalis* (fourth) *versus C. includens* (fourth)
	*A. gemmatalis* (isolated)
	*C. includens* (isolated)

^*^ Larval development: 4–12 h after ecdysis.

^*^ Adapted from Bentivenha *et al*.^39^

### 2.4 Statistical analyses

Fitness data (weight, survival, and cannibalism/predation) were submitted to analysis of variance, with normality and homoscedasticity using Shapiro–Wilk test and Levene's test, respectively.^40^ When differences occurred, Tukey's LSD's were reported at the *α* = 0.05 using PROC GLIMMIX procedure in SAS 9.4.^41^ The same procedure was used to analyze each of the five measurements of interest for the video‐tracking assay: distance moved (cm), distance between larvae (cm), body contact (s), time in food (s), and frequency in food (n). Residual and qq‐plots were used to assess normality. When normality assumptions were violated, a generalized linear mixed model (GLMM) was used to account for the underlying distribution of the data.

We analyzed the scenarios as a factorial treatment design depending on the response variable of interest and presence or absence of food. Distance moved was analyzed by a linear mixed model (LMM) with a 2 × 2 × 3 factorial treatment design: food – available/not available; treatment (species of larvae in which the distance moved was measured) – *A. gemmatalis/C. includens*; competitor – *A. gemmatalis/C. includens/isolated*. The variability due to the petri dish was considered as a random effect. Distance between larvae was analyzed using a linear model (LM) with a 2 × 3 factorial treatment design: food – available/not available; larvae combination – *A. gemmatalis + A. gemmatalis/A. gemmatalis + C. includens/C. includens + C. includens*. Body contact was analyzed as a generalized linear model (GLM) with a gamma distribution following the same 2 × 3 factorial treatment design as distance between larvae. Time in food and frequency in food were analyzed by generalized linear mixed models (GLMM) with a 2 × 3 factorial treatment design: treatment (species of larvae) – *A. gemmatalis/C. includens*; competitor – *A. gemmatalis/C. includens/isolated*. The variability due to the Petri dish was considered as a random effect. Time in food followed a gamma distribution while frequency in food followed a negative binomial distribution.

## 3 RESULTS

### 3.1 Fitness cost evaluation

Mean pupal weights of *A. gemmatalis* did not significantly differ among noncompeting and competing larvae surviving alone or in competition scenarios as third instar males (*F* = 2.46; df = 4, 23; *P* = 0.0744; Table 3), fifth instar males (*F* = 1.25; df = 4, 47; *P* = 0.3041), and fifth instar females (*F* = 0.39; df = 4, 26; *P* = 0.8148). Mean pupal weight of *A. gemmatalis* females from third instar larvae isolated (202.64 mg) was significantly greater than the pupal weight of small larvae surviving in competition (*F* = 2.91; df = 4, 20; *P* = 0.0476). The lowest means were observed when competing against fifth instar *A. gemmatalis* and fifth instar *C. includens*, with 130.85 mg and 145.65 mg, respectively. Regarding *C. includens*, we found no significant differences in pupal weight among different competition scenarios (*P ˃* 0.05; Table 3). However, in all cases and regardless of the species (*A. gemmatalis* or *C. includens*), higher values were observed in noncompeting scenarios while third instar larvae looked to be more negatively affected.

**Table 3:** Mean (± SE) pupal weight (mg) of *A. gemmatalis* and *C. includens* competing in different competition scenarios under laboratory conditions

Competition scenarios		Male		Female		
Treatment	Competitor	*n* *	Pupal weight (mg)†	*n* *	Pupal weight (mg)	
*A. gemmatalis* (third)	‐	8	201.46 ± 4.13	7	202.64 ± 12.43 a	
	*A. gemmatalis* (third)	8	178.84 ± 14.09	6	175.22 ± 15.46 ab	
	*A. gemmatalis* (fifth)	1	137.50 ± 0.00	2	130.85 ± 20.65 b	
	*C. includens* (third)	9	160.82 ± 11.03	4	176.73 ± 24.99 ab	
	*C. includens* (fifth)	2	165.80 ± 10.00	6	145.65 ± 9.15 b	
*P*			0.0744		0.0476	
*A. gemmatalis* (fifth)	‐	9	219.84 ± 4.92	6	224.33 ± 14.99
	*A. gemmatalis* (third)	9	203.93 ± 9.33	6	208.97 ± 13.41
	*A. gemmatalis* (fifth)	15	198.60 ± 6.27	9	201.24 ± 13.15
	*C. includens* (third)	10	203.51 ± 5.02	5	212.80 ± 13.19
	*C. includens* (fifth)	9	201.01 ± 10.20	5	214.78 ± 17.25
*P*			0.3041		0.8148	
*C. includens* (third)	‐	7	202.51 ± 11.46	8	193.38 ± 10.61
	*C. includens* (third)	11	174.84 ± 9.63	10	170.35 ± 14.23
	*C. includens* (fifth)	4	162.68 ± 20.84	5	162.90 ± 18.30
	*A. gemmatalis* (third)	1	159.80 ± 0.00	1	152.00 ± 0.00
	*A. gemmatalis* (fifth)	2	138.90 ± 28.40	3	131.87 ± 6.55
*P*			0.1476		0.2039	
*C. includens* (fifth)	‐	8	205.13 ± 9.41	7	211.33 ± 13.46
	*C. includens* (third)	5	199.76 ± 13.90	8	200.08 ± 10.61
	*C. includens* (fifth)	12	192.84 ± 12.39	13	193.96 ± 8.82
	*A. gemmatalis* (third)	4	194.25 ± 12.74	8	196.34 ± 7.92
	*A. gemmatalis* (fifth)	4	199.60 ± 6.32	6	201.18 ± 9.47
*P*			0.9472		0.7905	

* *n*, number of insects evaluated.

† Means followed by the same letter per column do not differ by Tukey's LSD test (*P* ˃ 0.05).

Larval survival of third instar *A. gemmatalis* (*F* = 10.72; df = 4, 70; *P* < 0.0001) and fifth instar (*F* = 3.40; df = 4, 70; *P* = 0.0134) was significantly affected by competition. The lowest survival means were observed on intraspecific scenarios involving fifth instar *A. gemmatalis* as a competitor, with 20.00% and 80.00% survival, respectively (Table 4). Regarding third instar *C. includens* (F = 11.13; df = 4, 70; *P* < 0.0001), the lowest survival means were related to interspecific scenarios, when the survival was 13.33% and 33.33% competing with third and fifth instar *A. gemmatalis* larvae, respectively. No differences occurred in scenarios involving *C. includens* fifth instar larvae (*F* = 1.72; df = 4, 70; *P* = 0.1545).

**Table 4:** Mean (± SE) larval survival (%), cannibalism/predation rate (%), and pupal survival (%) of *A. gemmatalis* and *C. includens* in different competition scenarios under laboratory conditions

Competition scenarios		*n* ^*^	Larval survival (%)†	Cannibalism/predation (%)	Pupal survival (%)
Treatment	Competitor				
*A. gemmatalis* (third)	‐	15	100.00 ± 0.00 a	‐	100.00 ± 0.00 a
	*A. gemmatalis* (third)	14	46.67 ± 10.31 c	10.00 ± 5.35 b	80.00 ± 11.06 ab
	*A. gemmatalis* (fifth)	3	20.00 ± 10.69 c	40.00 ± 13.09 a	0.00 ± 0.00 c
	*C. includens* (third)	13	86.67 ± 9.09 ab	0.00 ± 0.00 b	100.00 ± 0.00 a
	*C. includens* (fifth)	8	53.33 ± 13.33 bc	0.00 ± 0.00 b	75.00 ± 16.37 b
*P*			<0.0001	0.0004	<0.0001
*A. gemmatalis* (fifth)	‐	15	100.00 ± 0.00 a	‐	100.00 ± 0.00
	*A. gemmatalis* (third)	15	100.00 ± 0.00 a	0.00 ± 0.00	93.33 ± 6.67
	*A. gemmatalis* (fifth)	24	80.00 ± 8.16 b	6.67 ± 4.54	96.43 ± 3.57
	*C. includens* (third)	15	100.00 ± 0.00 a	0.00 ± 0.00	100.00 ± 0.00
	*C. includens* (fifth)	14	93.33 ± 6.67 ab	0.00 ± 0.00	92.86 ± 7.14
*P*			0.0134	0.1037	0.6846
*C. includens* (third)	‐	15	100.00 ± 0.00 a	‐	100.00 ± 0.00 a
	*C. includens* (third)	21	70.00 ± 9.51 ab	0.00 ± 0.00 b	88.46 ± 6.08 a
	*C. includens* (fifth)	9	60.00 ± 13.09 b	6.67 ± 6.67 b	77.78 ± 14.70 a
	*A. gemmatalis* (third)	2	13.33 ± 9.09 c	26.67 ± 11.82 ab	100.00 ± 0.00 a
	*A. gemmatalis* (fifth)	5	33.33 ± 12.60 bc	46.67 ± 13.33 a	40.00 ± 24.49 b
*P*			<0.0001	0.0042	0.0059
*C. includens* (fifth)	‐	15	100.00 ± 0.00	‐	100.00 ± 0.00 a
	*C. includens* (third)	13	86.67 ± 9.09	0.00 ± 0.00 b	100.00 ± 0.00 a
	*C. includens* (fifth)	25	83.33 ± 7.97	0.00 ± 0.00 b	100.00 ± 0.00 a
	*A. gemmatalis* (third)	12	80.00 ± 10.69	0.00 ± 0.00 b	83.33 ± 11.24 ab
	*A. gemmatalis* (fifth)	10	66.67 ± 12.60	20.00 ± 10.69 a	70.00 ± 15.28 b
*P*			0.1545	0.0212	0.0166

^*^ *n*, number of insects evaluated.

† Means followed by the same letter per column do not differ by Tukey's LSD test (*P* ˃ 0.05).

Third instar *A. gemmatalis* (*F* = 7.17; df = 3, 56; *P* = 0.0004) was affected by cannibalism (40.00%) when competing with fifth instar *A. gemmatalis* (Table 4). There was no difference in percentage of cannibalism/predation of fifth instar *A. gemmatalis* (*F* = 2.15; df = 3, 56; *P* = 0.1037) against the different competitors. In scenarios involving *C. includens*, 46.67% and 20.00% predation were observed for third (*F* = 4.91; df = 3, 56; *P* = 0.0042) and fifth (*F* = 3.50; df = 3, 56; *P* = 0.0212) instars in interspecific competition, respectively.

Differences in pupal survival were detected in scenarios with third instar *A. gemmatalis* (*F* = 12.27; df = 4, 44; *P* < 0.0001), third (*F* = 4.26; df = 4, 39; *P* = 0.0059) and fifth instar *C. includens* (F = 3.30; df = 4, 59; *P* = 0.0166) competing larvae (Table 4). In all the scenarios, the percentage of emerging adults was lower when larvae interacted with fifth instar *A. gemmatalis* as a competitor, except for fifth instar *A. gemmatalis* (*F* = 0.57; df = 4, 68; *P* = 0.6846), where no significant difference was observed when in intra or interspecific interactions.

### 3.2 Video tracking trials

**Figure 1:**
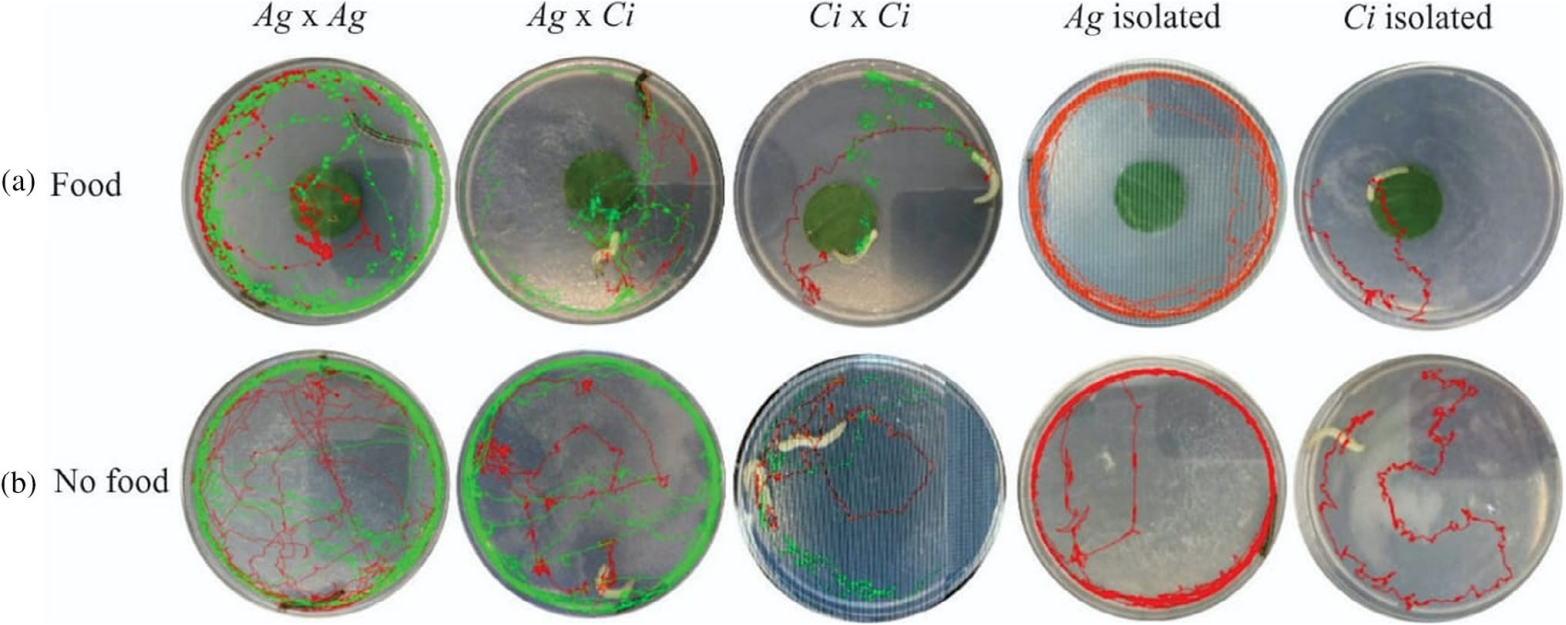
Representative movement of *A. gemmatalis* (*Ag*) and *C. includens* (*Ci*) larvae recorded over 20 min in different interaction scenarios with food and no food in glass petri dish arenas. For *Ag* x *Ag* and *Ci* x *Ci* scenarios, one larva track is red and the other green. For *Ag* x *Ci* scenarios, *Ag* is green, and *Ci* is red. For single larva scenarios, tracks are red.

The model response (ANOVA) results for each of the five response variables and associated model effects are in the Table 5. In addition to the main effects, model response for the distance moved indicated significant effects of the interactions between the factor's food × treatment and treatment × competitor at the *α* = 0.05 (Table 5). In conditions with food available, *A. gemmatalis* larvae moved a greater distance regardless of the competitor compared to *C. includens* larvae (*F =* 16.60; df = 5, 88*; P <* 0.0001) (Table 6; Fig. 1). Two *C. includens* larvae were closer together on average (3.66 cm) compared to two *A. gemmatalis* larvae (4.34 cm) (*F =* 3.16; df = 2,84; *P =* 0.0475), but they had less body contact (2.02 s) compared to two *A. gemmatalis* larvae or differing larvae species (*F =* 7.69; df = 2,84; *P =* 0.0009).

**Table 5:** Model response (ANOVA) variables and associated model effects from video tracking assay between *A. gemmatalis* and *C. includens*

Model effect	Distance moved	Distance between larvae	Body contact	Time in food	Frequency in food
Food	**20.67 (1, 88)**	**5.26 (1, 84)**	**12.15 (1, 84)**	‐	‐
Treatment	**256.06 (1, 88)**	‐	‐	1.36 (1, 44)	**19.41 (1, 44)**
Competitor	2.30 (2, 88)	‐	‐	2.29 (2, 44)	**4.68 (2, 44)**
Food × treatment	**16.86 (1, 88)**	‐	‐	‐	‐
Food × competitor	0.45 (2, 88)	‐	‐	‐	‐
Treatment × competitor	**4.32 (2, 88)**	‐	‐	0.54 (2, 44)	0.70 (2, 44)
Treatment × food × competitor	0.22 (2, 88)	‐	‐	‐	‐
Larvae combination	‐	**13.56 (2, 84)**	**5.31 (2, 84)**	‐	‐
Food × larvae combination	‐	1.51 (2, 84)	2.79 (2, 84)	‐	‐

ANOVA results are reported as *F*‐value (*df*). Significant model effects at the *α* = 0.05 level are indicated by a bold text.

**Table 6:** Mean (± SE) distance moved, distance between larvae, body contact, time in food, and frequency in food in scenarios with *A. gemmatalis* and *C. includens* interaction with food

		Food availability				
Treatment	Competitor	Distance moved (cm)*	Distance between larvae (cm)	Body contact (s)	Time in food (s)	Frequency in food (n)
*A. gemmatalis*	*A. gemmatalis* *C. includens* Isolated	256.33 ±19.88 a 245.57 ±24.50 a 198.78 ±24.50 a	4.34 ±0.19 a 3.86 ±0.19 ab ‐	15.84 ±6.70 a 14.99 ±6.34 a ‐	185.94 ±42.12 144.60 ±46.33 329.72 ±105.63	5.30 ±1.28 ab 8.79 ±2.73 a 2.27 ±0.79 abc
*C. includens*	*A. gemmatalis* *C. includens* Isolated	77.75 ±24.50 b 80.72 ±19.88 b 96.32 ±24.50 b	3.86 ±0.19 ab 3.66 ±0.19 b ‐	14.99 ±6.34 a 14.99 ±6.34 a 2.02 ±0.85 b ‐	208.01 ±66.64 268.97 ±60.93 365.49 ±117.09	1.99 ±0.70 bc 1.85 ±0.49 bc 0.96 ±0.38 c
*P*		<0.0001	0.0475	0.0009	0.25	0.0001

^*^ Means followed by the same letter per column do not differ by Tukey's LSD test (*P* > 0.05).

For time in food (Table 6), no difference was observed among the scenarios (*F =* 1.38; df = 5,44; *P =* 0.25). Regarding frequency in food, *A. gemmatalis* larvae were most frequently in food (8.79) when they were interacting with *C. includens* larvae, while *C. includens* larvae were least frequently in food (0.96) when they were isolated (*F =* 6.50; df = 5,44; *P =* 0.0001) (Table 6).

When there was no food present, *A. gemmatalis* larvae moved a greater distance when they were interacting with other larvae of either species compared to when *A. gemmatalis* were isolated and compared with larvae of *C. includens*, regardless of whether they were interacting with other larvae or isolated (*F =* 46.77; df = 5, 88; *P <* 0.0001). Two *A. gemmatalis* larvae were farther apart on average (5.01 cm) compared to any other scenario (*F =* 11.91; df = 2,84; *P <* 0.0001), while for body contact, no difference occurred among the scenarios (*F* = 0.40; df = 2,84; *P =* 0.67) (Table 7).

**Table 7:** Mean (***±*** SE) distance moved, distance between larvae, and body contact in scenarios with *A. gemmatalis* and *C. includens* interaction with no food

	No Food			
Treatment	Competitor	Distance moved (cm)*	Distance between larvae (cm)	Body contact (s)
*A. gemmatalis*	*A. gemmatalis* *C. includens* Isolated	379.29 ± 19.88 a 381.37 ± 24.50 ab 286.05 ± 24.50 b	5.01 ± 0.19 a 4.29 ± 0.19 b ‐	35.18 ± 14.89 24.05 ± 10.18 ‐
*C. includens*	*A. gemmatalis* *C. includens* Isolated	104.33 ± 24.50 c 89.06 ± 19.88 c 99.56 ± 24.50 c	4.29 ± 0.19 b 3.66 ± 0.19 b ‐	24.05 ± 10.18 21.00 ± 8.89 ‐
*P*		<0.0001	<0.0001	0.6719

^*^ Means followed by the same letter per column do not differ by Tukey's LSD test (*P* > 0.05).

## 4 DISCUSSION

In this study, we assessed for the first time the effect of competitiveness on development and behavior of *A. gemmatalis* and *C. includens*, two co‐occurring defoliating pests of soybean. These laboratory studies provide a clearer view of how interactions occur and specific behavior of the species under controlled conditions, which can be difficult to detect in the field where other factors such as environmental conditions, natural mortality, and disease might also influence the predominance of a species. Thus, this study provides baseline information, which can be useful for management of these important species in soybean in North and South America.

Results from the fitness study indicated that competition could impose a fitness cost, and the size of the competitor can impact insect development. Despite the male and female pupal weight factor not being affected, in general the survival in different stages was affected. The pupal weight of *A. gemmatalis* and *C. includens* in large larvae suggest that larvae did not suffer from food competition in advanced development stages. In general, there were no statistical differences observed in pupal weight, regardless of competitor and scenario, which is an important biological parameter and can impact the adult development, capacity of oviposition, offspring generated and the life history, in general.^42^

The survival of *A. gemmatalis* third instars competing in an intraspecific scenario with fifth instars was affected by the competition, presenting 80.00–100.00% mortality. Similarly, *C. includens* was negatively affected when competing against *A. gemmatalis* third and fifth instar. *Anticarsia gemmatalis* fifth instar had fewer effects to its development, with more than 80.00% and 92.00% larval and pupal survival, respectively. However, when *C. includens* fifth instar was in competition with others, larval and pupal survival were more affected, with 66.67% and 70.00% survival, respectively. Although food was available, the small surface area of the arena may have resulted in more encounters between the larvae, or not allowed escape from competitors. Furthermore, the relative density of resources within leaves could have differed and the small laboratory arena likely created an artificial scenario where another resource pool with higher protein was readily available, the competitor larvae in this case. In a situation where the superficial area is larger than the arena used in this study, and a larva has a high velocity of movement, the larva will likely have a greater probability to escape from cannibalism or predation.

In addition, this study did not evaluate larval attack or defense behaviors, actions of which are additional factors in the system, and may result in significant injury or death of individuals without direct killing from predation/cannibalism. These behaviors may have occurred in interspecific competition involving fifth instars *C. includens* and *A. gemmatalis*, where *C. includens* survival was affected, but just 20.00% predation was observed. Complementary ethogram studies examining larval attack and defense behaviors could clarify these results. Some theory indicates that larvae who end up practicing aggressive movements under naturalistic conditions, such as in the field, may have lower survival by suffering injuries and death, acquiring pathogens or parasites, and having subsequent reduction in fitness, lower pupal weight, and lower rates of development.^21^ Further, avoiding the other competitor could have incurred relatively substantial hormonal stress, depletion of lipids, missed feeding opportunities, or other indirect costs.^20^

Little is known about competition behaviors in intra or interspecific interactions involving *A. gemmatalis* and *C. includens*, but is well documented for other lepidopteran species.^32^, ^33^, ^39^, ^43^ In our interaction study under laboratory conditions, cannibalism and predation varied throughout the scenarios. Cannibalism occurred mainly in scenarios involving third *versus* fifth instar *A. gemmatalis* (40.00% observed). In addition, the highest predation rate was observed in interspecific competition between third instar *C. includens versus A. gemmatalis* fifth instar, and fifth *versus* fifth instar, with 46.67% and 20.00% respectively, suggesting that *A. gemmatalis* is more aggressive and could prevail over *C. includens*. Cannibalism and predation may be the most important mortality factors in insect populations,^44^ and several studies have showed that this behavior in different lepidopteran species is instar dependent, with higher rates related to delayed larval development, particularly when larvae of different instars are placed together and confined.^21^, ^33^, ^39^, ^45^, ^46^ However, there are other variables influencing the effect of competition resulting in aggressive behavior. Quality and quantity of food (*Bt* plant or not), population density, stress, and many other non‐consumptive effects and environmental factors (e.g., temperature and water availability) have impact on competition and fitness, specially under field conditions.^21^, ^22^

The results from the video tracking indicate that *A. gemmatalis* has a highly active behavior and tends to move more than *C. includens*. *Anticarsia gemmatalis* moves less when isolated than when interacting with another larvae, regardless of the species, confirming that the interaction influences larval behavior. This species moved constantly and mostly in a circle, repeatedly over the evaluation period (in food or no food scenarios). *Anticarsia gemmatalis* is more variable in how often it visits the food, depending on its interaction with the competitor, spending more time interacting with other larvae in scenarios with food. On the other hand, *C. includens* moved less and in an irregular pattern, visiting the food zone less often but spending more time there.

In general, the distance moved by *A. gemmatalis* was greater compared with *C. includens*, and this behavior might be one of the characteristic responses of larvae to escape an interaction.^45^, ^47^ Furthermore, due to the fact that it will be in constant movement it can result in more efficient chemical control of this species in non‐*Bt* soybean fields. In Brazil, *C. includens* larvae were observed to prefer to position themselves predominantly in the lower and mid region of the soybean plants,^48^ less exposed to insecticide, while the capacity of *A. gemmatalis* to move longer distances in the canopy may expose it more readily to treatment. Moreover, larval movement behavior and the difference in distance moved between the species emphasizes the importance of understanding on‐plant movement 49, 50 and plant‐to‐plant movement of lepidopterans.^51^, ^52^, ^53^ The movement of larvae in a seed mixture refuge strategy system, for example, might expose the insect to sublethal doses of *Bt* proteins by feeding on plant tissues of differential *Bt* protein content, or by initial feeding on a *Bt* plant and subsequent feeding on a non‐*Bt* plant, and *vice versa*, so larval mortality may not be achieved.^54^ Thus, the widespread release and adoption of *Bt* crops and the issues involving the different refuge models reinforce the necessity for further research to evaluate the effect of larval mobility and behavior on these strategies.

The distance between larvae in intra and interspecific scenarios involving *A. gemmatalis* was greater, which demonstrates that the species may have greater ability to escape from competitions. This species may have the habit of inciting aggressive behaviors, whether they are defensive or offensive mechanisms,^55^ and then, moving away from the competitor.^45^, ^47^ Associated with this, regarding body contact, *A. gemmatalis* spent more time interacting, both in intra or interspecific scenarios, and taking advantage over *C. includens*. In practical terms, this characteristic may be related to competition for food, increasing cannibalism and/or predation behaviors.

Regarding the frequency at food, the two species visited the food less when isolated than when interacting with other larvae, which was expected since the species would be competing for food in intraguild scenarios. For *Spodoptera frugiperda* and *Helicoverpa zea* (Lepidoptera: Noctuidae) in corn, the same behavior was observed.^39^ Although not statistically significant, both species spent more time in the food zone when isolated than when larvae were interacting with competitors, indicating that larvae might stop feeding or feed less when intraguild interactions occur. The decrease in larval feeding when larvae are interacting was described in a plant‐to‐plant movement study with *S. frugiperda*,^52^ and suggested that intraguild interactions could be related to larvae moving among plants, or may be a factor that increases the movement of larvae on alternative host plants. For insects in general, when there are restrictions in food availability and/or competitiveness, the outcome of the competition can be expressed more rapidly.^56^ Thus, as a secondary result, outbreaks of these species may also occur on alternative hosts, which are typically less preferred by the species, and the outcome of the competition may be more gradual or even uncertain.

Many animals will cannibalize as soon as all other food items are removed, but they may also respond simply to a reduction in the relative availability of alternatives, such as missed opportunity to feed or restrictions in food, leading to a food stress, and generally increasing food searching activity.^20^ Hunger triggers searching behavior, lowers the attack threshold, increases foraging time, and increases movement by stimulating locomotor activity, changing the location of foraging stations, and expanding the search area. Each of these behaviors increases the probability of intraspecific contact and predation.^20^ The differences in behavior in our study could be because the larvae were starved, the small size of the arena caused stress, or simply because of the competition for the soybean leaf (food availability scenario). In practical terms, and as a secondary result of the absence of food, outbreaks of these species may also occur on alternative hosts, which are typically less preferred by the species, and the outcome of the competition may be more gradual or even uncertain.

In general, based in the results, it is assumed that if these two species occur simultaneously on the same plant and in the same phenological phase, there is a greater chance of *A. gemmatalis* having an advantage. In summary, interactions among the leaf‐feeding Lepidoptera affect their behavior and our study showed that larvae of *A. gemmatalis* gain competitive advantage in intraguild interaction with *C. includens*. By using non‐*Bt* soybean, this study provides the baseline of larval behavior of two economically important species in intraguild interactions. The use of *Bt* technology is just one more factor in a complex system, which may impact population dynamics, pest prevalence, and the competitive displacement of species that share the same ecological niche. In the future it is necessary to explore fitness and behavior under field conditions where more factors are at play.

## 5 CONCLUSIONS

Our findings provide significant information regarding lepidopteran development and behavior, contributing to the development of integrated pest management and resistance management strategies of these species. More research is needed to fully understand the role of larval movement, feeding behavior, intraguild interaction focusing the non‐consumptive interactions , and other factors with respect to IPM and IRM.

## CONFLICT OF INTERESTS

All authors declare no conflict of interest.
